# Dynamics of 3D view invariance in monkey inferotemporal cortex

**DOI:** 10.1152/jn.00810.2014

**Published:** 2015-01-21

**Authors:** N. Apurva Ratan Murty, Sripati P. Arun

**Affiliations:** Centre for Neuroscience, Indian Institute of Science, Bangalore, India

**Keywords:** viewpoint invariance, object recognition, inferior temporal cortex

## Abstract

Rotations in depth are challenging for object vision because features can appear, disappear, be stretched or compressed. Yet we easily recognize objects across views. Are the underlying representations view invariant or dependent? This question has been intensely debated in human vision, but the neuronal representations remain poorly understood. Here, we show that for naturalistic objects, neurons in the monkey inferotemporal (IT) cortex undergo a dynamic transition in time, whereby they are initially sensitive to viewpoint and later encode view-invariant object identity. This transition depended on two aspects of object structure: it was strongest when objects foreshortened strongly across views and were similar to each other. View invariance in IT neurons was present even when objects were reduced to silhouettes, suggesting that it can arise through similarity between external contours of objects across views. Our results elucidate the viewpoint debate by showing that view invariance arises dynamically in IT neurons out of a representation that is initially view dependent.

object vision is challenging because images of an object vary widely with the viewing conditions (Dicarlo et al. 2012; Logothetis and Sheinberg 1996; Pinto et al. 2008; Poggio and Ullman 2013; Tanaka 1996). Rotations in depth can pose a particular challenge because features can appear, disappear, be stretched or compressed. Yet we easily recognize most natural objects across rotations in depth. Is the underlying object representation view dependent or invariant? This question has been extensively debated in human vision. While judging whether two images belong to the same object, humans take longer to respond to disparate views. This has been taken as evidence for viewpoint dependence (Andresen et al. 2009; Fang and He 2005; Gauthier et al. 2002; Hayward and Tarr 1997; Tarr 1995; Tarr et al. 1997, 1998; Tarr and Buelthoff 1995). However, these effects are mitigated or even absent for dissimilar objects (Edelman 1995; Foster and Gilson 2002; Lawson and Buelthoff 2006, 2008; Newell 1998; Wilson and Farah 2003). Biederman and colleagues have argued that objects that differ in nonaccidental properties exhibit viewpoint invariance, whereas objects differ in metric properties exhibit viewpoint dependence (Biederman and Cooper 1991; Biederman and Gerhardstein 1993, 1995). These findings suggest that view dependence and invariance depend on object structure and may even coexist (Foster and Gilson 2002; Lawson and Buelthoff 2006, 2008; Stankiewicz 2002). However, precisely which image properties determine view dependence and invariance remain unclear.

Despite these insights from human studies, the underlying neuronal representations have received relatively little attention. A natural locus for view-invariant representations is the monkey inferotemporal (IT) cortex, whose neurons are invariant to attributes such as size, position and clutter (Dicarlo et al. 2012; Tanaka 1996). However, there are mixed reports of view invariance in IT neurons. On the one hand, IT neurons respond to some but not all views of artificial objects in naive animals (Yamane et al. 2008). View-specific responses have also been observed for wireframe objects, despite extensive training on view-invariant recognition (Logothetis et al. 1994, 1995; Logothetis and Pauls 1995). Such responses can delay the process of matching the views of an object and can give rise to view dependence in behavior (Perrett et al. 1998). On the other hand, IT neurons respond in a view-invariant manner to familiarized natural objects (Booth and Rolls 1998) and faces (Eifuku et al. 2004, 2011; Freiwald and Tsao 2010; Perrett et al. 1991). These disparate findings can be reconciled by the fact that the first set of studies used artificial objects, such as wireframes, whose images vary drastically with viewpoint, whereas the second set of studies used natural objects whose images do not vary greatly with viewpoint. Therefore, it is not clear whether IT neurons are view invariant for naturalistic objects in general. To elucidate this issue, we systematically tested IT neurons using naturalistic objects in monkeys with no prior training on these objects.

We performed a total of three experiments. In *experiment 1*, we recorded neuronal responses to a diverse set of objects in two views each. Here, we observed a dramatic transition wherein neurons were initially strongly modulated by view and subsequently were modulated by object identity. This transition was strongly dependent on object structure: it was strongest when objects foreshortened strongly across views and were similar to each other at the pixel level. In *experiment 2*, we confirmed that this transition was present even for objects across multiple views. In *experiment 3*, we compared view invariance for objects and their silhouettes. Here, view invariance was still present, suggesting that invariance can arise through similarity between external contours across views. Finally, we tested a number of computational models for their ability to predict the view invariance observed in our data. The early sensitivity to view in IT was explained by low-level pixel differences, whereas the later view invariance was partly explained by affine matching operations between object views. Our results suggest a coarse-to-fine processing scheme in IT, wherein neurons are initially sensitive to coarse, low-level differences between views and only later become invariant to object identity independent of view.

## METHODS

All experiments were performed according to a protocol approved by the Institutional Animal Ethics Committee of the Indian Institute of Science, Bangalore and the Committee for the Purpose of Control and Supervision of Experiments of Animals, Government of India. These procedures also conform to the APS Guiding Principles in the Care and Use of Vertebrate Animals in Research.

### Neurophysiology

**Fig. 1.**
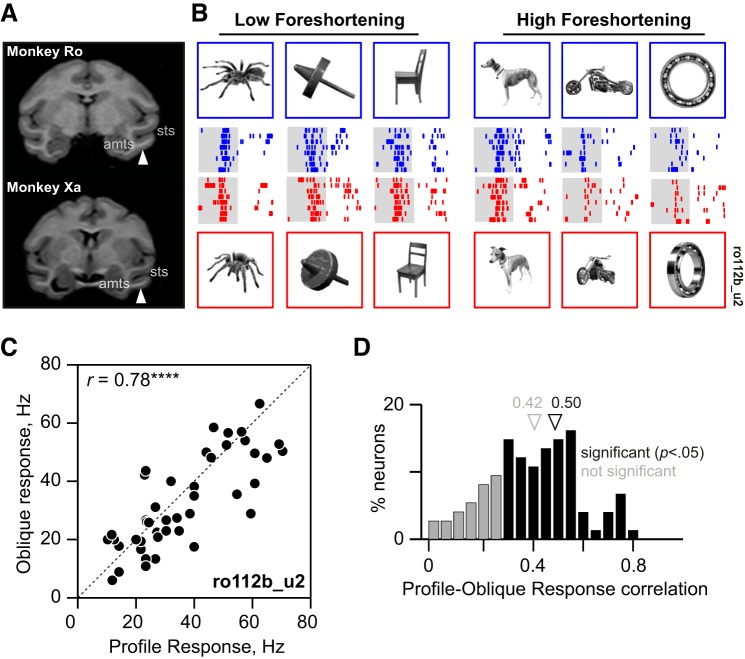
Three-dimensional view invariance in inferotemporal (IT) neurons. *A*: coronal slices from structural MRI in each monkey (Ro and Xa) at a position along the anterior-posterior axis corresponding to the center of their respective recording chambers. The white arrow represents the approximate location in IT targeted by the center of the recording grid. Anatomical landmarks are indicated for reference (sts, superior temporal sulcus; amts, anterior medial temporal sulcus). *B*: responses of a single IT neuron to profile and oblique views of a few example objects (out of the full set of 45). *Top* row: profile views and their responses (blue rasters; each row is one trial, with ticks marking spike times). *Bottom* row: corresponding oblique views and their responses (red rasters). The shaded area is the 200-ms period for which the image was displayed. In the experiment, images were presented against a black background. *C*: responses to the oblique view plotted against the response to the profile view for all 45 objects in the experiment for the example neuron. Here and in all following figures, asterisks represent statistical significance [not signficant (n.s.) is *P* > 0.05, *P < 0.05, **P < 0.005, etc.]. *D*: distribution of profile-oblique correlations across the neuronal population (*n* = 111 neurons). Black bars indicate statistically significant correlations (*n* = 84; *P* < 0.05), and gray bars indicate the nonsignificant ones (*n* = 27; *P* > 0.05). [Object pictures from Amsterdam Library of Object Images (Geusebroek et al. 2005), reprinted with permission; or Hemera Photo Objects, reprinted with permission, copyright 2015, S. P. Arun and licensors, all rights reserved.]

Two adult male monkeys (*Macaca radiata*, laboratory designations Ro and Xa, both aged 14 yr, colony-born) were used in the study. Prior to recording, each monkey was surgically implanted with a titanium head-post and a Cilux recording chamber (Crist Instruments). The recording chamber was guided through structural MRI to be centered on the anterior half of IT, i.e., lateral to the anterior medial temporal sulcus on the ventral bank of the superior temporal sulcus (STS) (Fig. 1*A*). The center of the recording chamber was placed at a location which corresponded to 18 mm anterior to the interaural plane in a standard rhesus monkey anatomical atlas (Saleem and Logothetis 2007); this corresponded to anterior 14 mm and lateral 19 mm in monkey Ro and anterior 18 mm and lateral 18 mm in monkey Xa (Fig. 1*A*). This was subsequently verified in the latter using postmortem histology. Eye position was monitored using an infrared eye tracker system (ISCAN). On each day of recording, a tungsten microelectrode with an initial impedance of about 1 MΩ at 1 kHz (FHC) was inserted using a square grid with 1-mm spacing through a transdural guide tube and advanced to ∼10 mm above IT. The electrode was then lowered using a micromanipulator (Narishige) until phasic visual responses were observed. Action potentials were isolated from the multiunit trace after high-pass filtering (4-pole Butterworth filter, cutoff frequency 75 Hz) using a commercial spike sorting system (Plexon), and these waveforms were sorted into individual units afterwards based on cluster and waveform analysis (Offline Sorter, Plexon). Stimulus presentation, reward and eye position were under control of a computer running Cortex software (National Institute of Mental Health DOS Cortex; 120-Hz Viewsonic monitor).

### Behavioral Task

Neuronal activity was recorded during passive fixation. The monkey was seated at a distance of 50 cm from a computer monitor (Viewsonic VX2268wm, 120-Hz refresh rate). On each trial, a red fixation cross (measuring 0.6°) appeared at the beginning, and the animal was required to maintain its gaze within a 3° rectangular window centered on the cross. About 200 ms after attaining fixation, eight images appeared for 200 ms each with an interstimulus interval of 200 ms. The monkey received a juice reward for successfully maintaining fixation throughout the trial. Although the fixation window was large, post hoc analyses of the monkeys' eye movements revealed that gaze remained closely centered on the cross throughout the trial (average standard deviations: 0.33° along horizontal, 0.24° along the vertical).

### Experiment 1 (Objects at Two Views)

A total of 90 gray-scale images were used in the main experiment. We avoided showing full-color objects to preclude finding an artifactual view invariance arising from neuronal selectivity for color. We used a total of 45 naturalistic objects: 15 animals (including four-legged animals, birds and insects) and 30 inanimate man-made objects chosen from Hemera Photo Objects and the Amsterdam Library of Object Images (Geusebroek et al. 2005). We selected images corresponding to two views: a profile view (defined as the view in which the object was elongated), and an oblique view that corresponded to roughly 45° rotation in depth, i.e., out of the image plane. Profile views were scaled uniformly such that the longer dimension subtended a visual angle of 5.5°. Each oblique view was scaled such that its height matched the height of the profile view, with the result that it appeared to be a plausible depth-rotated version of the profile view.

#### Trial design.

Stimuli were separated into two groups of 24 each: one containing 17 animals and 7 things, and the other containing 24 things. Three objects (2 animals, 1 thing) were subsequently discarded because their views were erroneously selected from different objects. Each trial consisted of eight stimuli presented for 200 ms with a 200-ms interstimulus interval. Stimuli were presented in random order. On a given trial, stimuli alternated between the two groups (with either group appearing first) and between profile and oblique views with the constraint that the two views of the same object never occur in a single trial. Each stimulus was repeated a total of eight times. A total of 111 visually responsive neurons (*n* = 85 from monkey Ro, *n* = 26 from monkey Xa) were recorded from 58 sites in this experiment.

### Experiment 2 (Multiple Views)

A total of six objects were used in this experiment (dog, elephant, fish, gun, motorbike, trumpet). Each object was presented in seven views. For each object, we took the profile view (i.e., the view at which its image was the most elongated, scaled to 5.5° as before) to be 0°, and the six views corresponded to rotations of 30, 60, 75, 90, 105, 120 and 150° in depth. Each trial consisted of seven stimuli presented for 200 ms, each with a 200-ms blank interval. Stimuli were presented in random order, with the constraint that all stimuli presented in each trial belonged to distinct objects. For the purposes of this study, we removed the 90° view from all analyses because it severely obscured object identity. Each stimulus was repeated a total of eight times across trials. A total of 23 visually responsive neurons (*n* = 16 from monkey Ro, *n* = 7 from monkey Xa) were recorded from 16 sites in this experiment.

### Experiment 3 (Silhouettes)

The stimuli in this experiment consisted of 24 objects (12 animals and 12 things) chosen from the objects in *experiment 1* (see Fig. 7*A*). The two views of each object were shown in natural and silhouette forms. The two versions were run in separate blocks, with block order chosen at random for each session. Other details, such as repetitions and stimulus order in a trial, were as in *experiment 1*. A total of 61 visually responsive neurons (*n* = 27 from monkey Ro, *n* = 34 from monkey Xa) from 38 sites were recorded in this experiment.

### Data Analyses

#### Analysis of variance.

The stimuli in *experiment 1* consisted of 45 objects presented in two views each. To assess the degree to which each neuron was modulated by object identity or by view, we analyzed neuronal responses to two objects at two views each so that object and view effects could be compared in an unbiased manner. For neuron and object pair (A, B), we performed an ANOVA on the firing rates with view (profile/oblique) and object (A/B) as factors. To measure the effect strength, we calculated the difference between the response to the preferred and nonpreferred conditions. For instance, the object main effect strength is the difference between the responses to the preferred and nonpreferred objects. Likewise, the view main effect strength is the response difference between the preferred and nonpreferred views.

A direct measure of object effect strength would be the difference between responses to preferred and nonpreferred objects. However, this measure is biased because it will always yield a positive difference even for random noise. To overcome this limitation, we used a split-half approach. Using the odd-numbered trials, we identified the preferred object (across views) and then used the even-numbered trials to calculate the difference between the responses to the preferred and nonpreferred objects. This process was repeated by identifying the preferred object using the even-numbered trials and calculating the response difference using the odd-numbered trials. These two estimates were averaged together to yield an unbiased estimate of the effect strength. This method ensures that the average effect strength is zero for random noise. We used the same approach to calculated unbiased estimates of view and interaction effect strengths.

#### Baseline view invariance index.

To assess the baseline level of view invariance present in the image, we calculated a modulation index of the form (O − V)/(O + V), where O is the object modulation, calculated as the summed absolute difference in pixel intensities between the two objects averaged across views, and V is the view modulation, calculated as the summed absolute difference in pixel intensities between two views, averaged across objects. A baseline view invariance of 1 would indicate strong object modulation and weak view modulation, as may be expected from two objects, such as a wine glass and a sphere that are symmetric about rotations in depth. A baseline view invariance of −1 would indicate strong view modulation and weak object modulation, as may be expected for highly similar objects that foreshorten greatly across views. To compare this with the view invariance in IT, we calculated an analogous view invariance index for each object pair using the peak object effect strength and the peak view effect strength (averaged across neurons).

#### Population decoding.

For each trial of every image and in each time bin, we calculated a population response vector containing the firing rates evoked by all neurons. This analysis assumes that neuronal responses were recorded simultaneously, whereas they were in fact recorded on separate sessions. The resulting estimate of decoding accuracy is a lower bound on the information present in the population (Hung et al. 2005). We projected the entire set of response vectors along their principal components to retain 80% of the total variance. This step was necessary, particularly for the initial time bins when many neurons produce a zero response and consequently infinitely many solutions for the best hyperplane separating the classes. Varying the fraction of retained variance did not qualitatively change any of the results. We then trained multiclass linear classifiers (*classify* function in Matlab) on the response vector and the associated label (for the view decoder, a binary vector representing the profile or oblique view; for the object decoders, a vector of integers representing object identity). We evaluated the performance of the view decoder using a leave-one-out procedure: for each trial, we trained the decoder on the remaining trials and predicted its view label. On repeating this for all trials, we obtained a percent-correct measure that indicates how well view identity could be decoded using the population responses in that trial. We used an analogous procedure for the view-specific object decoder: we used a leave-one-out process to obtain the predicted object label at each view, and averaged the performance across views. To evaluate the view-invariant object decoder, we trained the decoder only on responses to one view (e.g., profile) and tested it on trials in which the other view was shown (e.g., oblique). This was done with both views as the training data and the resulting performance were averaged to obtain an estimate of view-invariant object decoding.

In *experiment 3* (multiple views), we used a similar approach. For the view decoder, we used a leave-one-out procedure to obtain a predicted view label for each trial. For the view-specific object decoder, we trained a multiclass object classifier at each view, evaluated it using a leave-one-out procedure, and averaged the performance across views to get an estimate of view-specific object decoding. For the view-invariant object decoder, we trained a multiclass object classifier on all trials, regardless of view, and evaluated it using a leave-one-out procedure. We obtained qualitatively similar results on evaluating all three decoders using pairs of views instead of all views at once and observed no systematic variations in our results with differences in view angle.

#### Multidimensional scaling.

To visualize similarity relations between objects across the neuronal population, we used multidimensional scaling. We calculated the similarity in neuronal activity elicited for every pair of images as the average absolute difference in firing rate elicited by these images across neurons (other measures of distance, such as correlation or Euclidean metrics, yielded similar results). This method finds the coordinates of each image in a two-dimensional space such that the distances between images best match the pairwise neuronal distances. This was implemented using standard functions (*mdscale*, MATLAB). To assess the degree of match between the two-dimensional embedding and the actual neuronal distances, we calculated the correlation between them. This correlation coefficient is shown in each multidimensional scaling plot and was generally high, indicating that the multidimensional scaling plots are a reasonable approximation to the observed similarity relations.

### Computational Models

#### V1 model.

To investigate whether the observed similarity data could arise from neural activity in V1, we set up a standard V1 model consisting of Gabor filters (Pinto et al. 2008). For every image, the V1 model produces the responses of a population of putative V1 neurons using a bank of Gabor filters with lateral interactions that implement divisive normalization. In the V1 model, we used a scaling factor of 1 dva = 18 pixels based on published observations regarding the sizes of V1 receptive fields. Our V1 model was identical to a previously published model (Pinto et al. 2008). The output of the V1 model consisted of the activity of 96 model units in response to an image, which was concatenated to create a feature vector.

#### HMAX model.

We evaluated a model of high-level visual cortex (without training on our stimuli), for its ability to match the observed view dependence and invariance in IT. The model has several layers of units (S1, C1, S2 and C2), which alternate between summing and winner-take-all (MAX) computations. We used an implementation of the HMAX model from a previous study (Riesenhuber and Poggio 2000; downloaded in October 2013 from http://riesenhuberlab.neuro.georgetown.edu, now available at http://cbcl.mit.edu/software-datasets/standardmodel/index.html), with parameters based on a recent report from the same group (Serre and Riesenhuber 2004). In the implementation, the first layer (S1) can be chosen to have orientation tuning with Gaussian, Gabor or Difference-of-Gaussian functions. Among the three filters, the Gaussian filter yielded the best fits, which are what we report here. We chose the C2 responses because they are the most complex and, therefore, most likely to show view invariance. For each image, the model returns the response of 256 C2 units, which we took as the feature vector.

## RESULTS

We performed three experiments to investigate the dynamics of view invariance in IT neurons. In all experiments, monkeys were trained to fixate a series of images presented at the fovea and had no prior visual experience or training on these images.

Following previous studies, we measured view invariance by comparing the modulation across views of an object with the modulation across object identity. However, it is important to compare the view invariance measured in IT neurons with that expected from the image itself. As an extreme example, consider a wine glass and a sphere. Both objects produce the same image when rotated in depth, resulting trivially in complete view invariance. To establish a baseline level of invariance, we compared the average change in pixels across views with the average change in pixels across objects. In general, a horizontally elongated planar object (e.g., motorbike) will produce greater image changes across views than a round nonplanar object (e.g., kettle) because it foreshortens strongly. Thus the baseline level of view invariance will be small for objects that foreshorten strongly across views. We selected a diverse set of objects to investigate the specific image properties that influence view invariance and its dynamics.

### Experiment 1: Objects in Two Views

Here, we recorded the activity of 111 neurons from the left anterior IT cortex of two monkeys (Fig. 1*A*). We chose a large number of objects at the expense of views to activate a broad range of neurons. In all, there were 45 objects (15 animate and 30 inanimate objects), each presented in two views. For each object, the view in which it was most elongated was defined as the profile view. The other view, defined as the oblique view, corresponded to a 45° rotation in depth about the vertical axis from the profile view. We chose 45° rotations as a reasonable compromise between smaller rotations that produce highly similar images and larger rotations that potentially obscure object identity (Blanz et al. 1999; Gomez et al. 2008) or lead to mirror confusion (Rollenhagen and Olson 2000).

Figure 1*B* shows the responses of a single IT neuron to a subset of the objects at both views. Its responses were invariant across views, as evidenced by a high correlation between the firing rates evoked during the image presentation period (0–200 ms) across all 45 objects (*r* = 0.79, *P* < 0.0005; Fig. 1*C*). This view invariance was extremely widespread across the population: 84 of the 111 neurons (76%) exhibited a significant response correlation (*P* < 0.05) between the two views (average correlation: *r* = 0.42 ± 0.18; average significant correlation: *r* = 0.50 ± 0.13; Fig. 1*D*).

We observed no significant differences in this correlation between subgroups of objects (*r* = 0.28 for animates, *r* = 0.32 for inanimates; *P* = 0.24, Wilcoxon signed-rank test). There was also no significant tendency for view-invariant neurons to be located at more anterior or lateral recording sites (response correlation vs. recording location: along the anterior-posterior axis: *r* = −0.15, *P* = 0.13; along the medial-lateral axis: *r* = −0.06, *P* = 0.54). To assess whether invariant neurons have longer response latencies, we investigated the relationship between the response latency (measured as the time at which the average firing rate attained its peak) for each neuron and its response correlation across views. We found no significant tendency for view-invariant neurons to have delayed responses (i.e., there was no relationship between profile-oblique correlation and peak response latency across neurons: *r* = 0.06, *P* = 0.42; average peak latency: 109 ms).

### Object and View Modulation in IT Neurons

To investigate view invariance in greater detail, we characterized the degree to which object identity and view modulated the neuronal response. A neuron that is view invariant would be strongly modulated by object identity and less so by view, whereas a neuron that is view dependent would show the opposite pattern. Although in principle we could have performed an ANOVA with object identity (45 objects) and view (2 views) as factors, the larger number of objects compared with views could potentially overestimate object identity effects compared with view effects simply because of wider sampling. To avoid this problem, we performed ANOVAs on pairs of objects with two views each. This allowed us to compare in an unbiased manner the strength and incidence of view and object identity effects across neurons and objects. Of particular interest to us was the incidence of main effects of object identity that occurred without a significant view or interaction effect, indicative of view invariance.

**Fig. 2.**
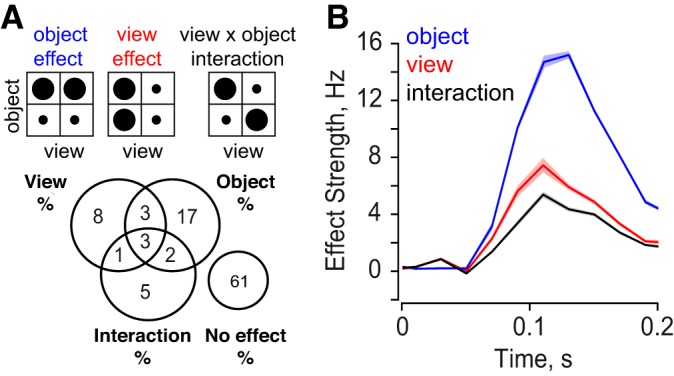
View and object modulation in IT neurons. *A*: for each pair of objects in both views, we performed an ANOVA with object and view as factors (see text). *Top*: schematic of object, view and interaction effects, with circle size representing the magnitude of response. *Bottom*: percentage of significant effects (*P* < 0.05) observed across cells and object pairs. The most frequent effect was that of a pure object main effect (17%), indicative of view invariance. The cases with no effect consisted of stimuli which elicited responses below spontaneous levels. *B*: time course of object (blue), view (red) and interaction effects (black) in 20-ms bins during the image presentation period (200 ms). In each case, the effect strength is calculated as the difference between preferred and nonpreferred responses. Shaded regions represent standard errors of mean calculated across all neurons and pairs. The high incidence and magnitude of object main effects indicate the widespread view invariance observed in IT neurons.

We performed an ANOVA on the firing rates of each cell (during 0–200 ms after image onset) to pairs of objects (A, B) in both views, with view (profile/oblique) and object identity (A/B) as factors. Across all 111 cells and all 990 pairs of objects (^45^C_2_), we observed 1.6 times as many main effects of object identity (25% of cases) as for view (15% of cases) (Fig. 2*A*). Importantly, among the cases with an object main effect, 68% of them had no other significant view or interaction effect, indicating view invariance (Fig. 2*A*). To investigate whether view and object identity modulation co-occurred across neurons, we compared the number of significant view effects against the number of significant object identity effects for each neuron. We found a significant correlation between these two measures (*r* = 0.6, *P* < 0.00005). Thus neurons that show strong object identity modulation also show strong view modulation.

To investigate the temporal evolution of these signals, we obtained unbiased estimates of the object identity, view and interaction effect strengths in 20-ms time bins across the entire visual response (see methods). Across all cells and object pairs, object identity signals were consistently twice as large in magnitude compared with view signals (Fig. 2*B*). Thus IT neurons are modulated more by object identity than by view.

### Dynamics of Object and View Signals Depend on Object Structure

We then asked whether the dynamics of view and object identity vary with object structure. We tested two specific predictions: *1*) objects that foreshorten strongly with rotation exhibit greater image changes across views and should therefore elicit stronger view modulation; and *2*) objects that are dissimilar should have greater object modulation since their images are more distinct at the pixel level.

To measure the foreshortening experienced by each object with changes in view, we calculated the decrease in its width across views relative to its width in the profile view. In general, this measure will be small for nonplanar objects in which new surfaces become visible with rotation in depth, and large for planar objects which foreshorten strongly upon rotation. Among the many possibilities we tested, this measure of foreshortening was best able to explain the observed view modulation in IT neurons. For instance, other measures of low-level differences, such as the summed pixelwise difference between the two views, did not account as well for the observed view modulation in IT neurons.

**Fig. 3.**
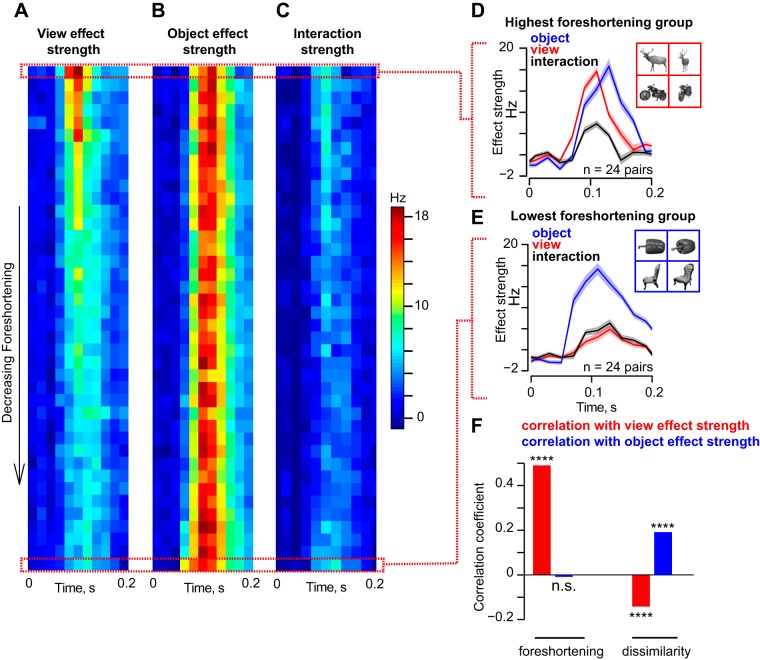
Dynamics of view and object modulation in IT neurons. *A*: for each pair of objects, we calculated the strength of the view main effect as a function of time during the entire image presentation period (200 ms). We then sorted all 990 pairs (^45^C_2_) in order of decreasing foreshortening and averaged the time course across groups of ∼24 pairs each (2.5% of the entire set). The resulting time courses for each group of objects are shown as a colormap. *B*: time course of object main effect strength for the same object pairs as before. *C*: time course of interaction effect strengths for the same object pairs as before. *D*: time course of view, object and interaction effects for object pairs with the highest foreshortening (*n* = 24). An example object pair (deer, motorcycle) is shown in the *inset*. *E*: time course of view, object and interaction effects for object pairs with the lowest foreshortening (*n* = 24), with an example object pair (pepper and chair). *F*, *left*: correlation (across all 990 pairs) between image foreshortening and peak view and object strengths. Foreshortening increased view modulation but did not influence object modulation. *Right*: correlation between image dissimilarity and peak view and object strengths. [Object pictures from Hemera Photo Objects. Reprinted with permission. Copyright 2015, S. P. Arun and licensors. All rights reserved.]

To investigate how foreshortening affects view and object modulation in IT, we sorted object pairs in descending order of average foreshortening and plotted the time course of the object identity, view and interaction effects as a colormap (Fig. 3, *A–C*). We found a striking difference in dynamics: for objects with high foreshortening, such as the deer and motorbike (Fig. 3*D*, *inset*), view modulation was strong and peaked before object identity modulation (Fig. 3*D*). Thus there is a transition in the neuronal response from a representation dominated by early viewpoint dependence (i.e., modulation across views) to a representation dominated by viewpoint invariance (i.e., modulation by object identity) for high-foreshortening objects. These dynamics were different for objects with low foreshortening, such as the pepper and chair (Fig. 3*E*, *inset*). Here, we observed an early and strong object identity modulation that persisted throughout the visual response (Fig. 3*E*). To investigate how view modulation is influenced by foreshortening, we calculated the correlation between the peak view modulation and the average foreshortening across object pairs. This yielded a significant positive correlation (*r* = 0.52, *P* < 0.0005, Fig. 3*F*). However, the strength of object modulation did not vary systematically with foreshortening (*r* = −0.01, *P* = 0.72, Fig. 3*F*).

Our second prediction was that dissimilar objects would exhibit greater view invariance because they remain distinct across views. To assess this possibility, we measured for each object pair the summed absolute difference between pixel intensities for profile and oblique views separately, and averaged this across views. This yields a measure of pixel-level dissimilarity between objects. Across object pairs, pixel dissimilarity was negatively correlated with peak view modulation (*r* = −0.16, *P* < 0.0005), and positively with peak object modulation (*r* = 0.2, *P* < 0.0005; Fig. 3*F*). Thus dissimilar objects tended to show increased object identity modulation and decreased view modulation.

We also assessed how object × view interactions are influenced by foreshortening and dissimilarity in a similar fashion. The peak interaction strength was weakly correlated with foreshortening (*r* = 0.11, *P* < 0.0005) and with dissimilarity (*r* = 0.08, *P* < 0.05).

In summary, we conclude that dynamics of view dependence (modulation across views) and view invariance (modulation across objects) in IT neurons are influenced by two specific image attributes: foreshortening and dissimilarity.

### How Much View Invariance Is Present in the Retinal Image?

**Fig. 4.**
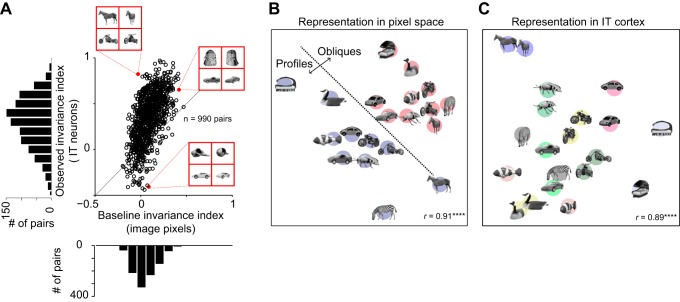
View invariance in IT compared with baseline view invariance. *A*: for each pair of objects, we calculated a baseline view invariance index which measures the view invariance present directly in the image pixels. In the plot, the observed view invariance index in IT is plotted against the baseline view invariance from pixels. *B*: similarity relations for a set of objects in pixel space obtained using multidimensional scaling. In pixel space, objects exhibit view dependence i.e., profile and oblique views tend to cluster together. *C*: similarity relations for the same set of objects in IT neurons. In IT neuronal space, objects exhibit view invariance i.e., objects tend to cluster despite changes in view. ****Statistical significance (*P* < 0.00005). [Object pictures from Amsterdam Library of Object Images (Geusebroek et al. 2005), reprinted with permission; or Hemera Photo Objects, reprinted with permission, copyright 2015, S. P. Arun and licensors, all rights reserved.]

In the above analyses, we have compared view and object modulation for a set of naturalistic objects across two views and found that IT neurons are modulated stronger by object identity than by changes in view. However, changes in view and object are fundamentally different changes, so we sought to establish a common baseline. To this end, for each pair of objects, we devised a baseline invariance index, which measures the net change in image pixels due to changes in object relative to the net change due to changes in view (See methods). Objects that are symmetric about the rotation axis, such as the wine glass and sphere example discussed earlier, will produce a baseline invariance close to 1, indicating perfect view invariance in the image itself. At the other extreme, objects that are extremely similar to each other but whose images change across views will produce a baseline invariance close to −1, indicating extreme view dependence. We then compared this baseline invariance with an analogous measure calculated for IT neurons using the peak view and object effect strengths (Fig. 4*A*). The observed view invariance was significantly correlated with the baseline view invariance (*r* = 0.6, *P* < 0.00005); however, this is only expected because a high baseline view invariance implies objects whose images change little across views, resulting in similar responses in IT neurons to both views and consequently a large view invariance index. Importantly, however, the view invariance in IT was far stronger than the invariance present in the retinal image (average invariance index across 990 pairs: 0.09 for image pixels, 0.34 for IT neurons; *P* < 0.0005, Wilcoxon signed-rank test). To be sure that this result was not due to differences in the magnitude of numbers used (firing rates vs. pixel intensity), we calculated the number of view-invariant object pairs in each case, defined as the pairs with greater object modulation compared with view modulation. The fraction of view-invariant pairs was also significantly greater in IT compared with baseline (percent significant pairs: 89% in IT vs. 74% in baseline; *P* < 0.001, χ^2^ test).

To visualize how objects and their views are arranged in pixel space, we performed a multidimensional scaling analysis on all pairwise pixel distances between a subset of the objects. In this analysis, images with a large pixel distance are placed far away, and similar images are placed closer. The resulting plot (Fig. 4*B*) shows that, in the image pixels, profile and oblique views form distinct clusters, indicative of view dependence. To compare this with the IT representation, we performed a similar multidimensional scaling analysis on pairwise neuronal distances (calculated as the average absolute difference in firing rate across neurons). The resulting plot shows that the IT representation is view invariant (Fig. 4*C*).

We conclude that the view invariance in IT is stronger than expected from the retinal image. The fact that object identity signals develop over time also implies that view invariance is a consequence of computations either within or presynaptic to IT.

### Population Read-out of View and Object Identity

The above results show that the dynamics of object and view modulations are influenced by object structure, but are based on analyzing responses of single neurons to pairs of objects. To test view invariance across larger groups of objects, we asked how well the response of the entire neuronal population could be used to infer object identity and object view on a trial-by-trial basis. We decoded two types of object information: we measured decoding of object identity at each view (view-specific object decoding), and the decoding of object identity invariant of view (view-invariant object decoding).

To measure view decoding, we measured the performance of a linear classifier trained on single trials with a view label (profile/oblique) while ignoring object identity. Note that the ability to decode view, regardless of object, is most likely because profile views are generally wider than oblique views as such, and not because of any viewpoint encoding per se. This is consistent with our earlier finding that view modulation is correlated with image foreshortening (Fig. 3) and with the finding detailed in a later section that view-clustering is explained by a pixel model (see Fig. 8). To measure view-specific object decoding, we used a linear classifier trained on all trials corresponding to a single view with the corresponding object labels. To measure view-invariant object decoding, we trained a linear classifier on responses to profile views and tested it on responses to oblique views (or vice versa). All three classifiers are described in detail in the methods. We performed this decoding analysis on neuronal responses in 20-ms time bins over the entire image presentation period (200 ms). Note that, because the number of views and objects are different, decoding of view cannot be compared with decoding of view-specific or view-invariant object identity in terms of magnitude, but can only be compared in terms of their temporal dynamics.

**Fig. 5.**
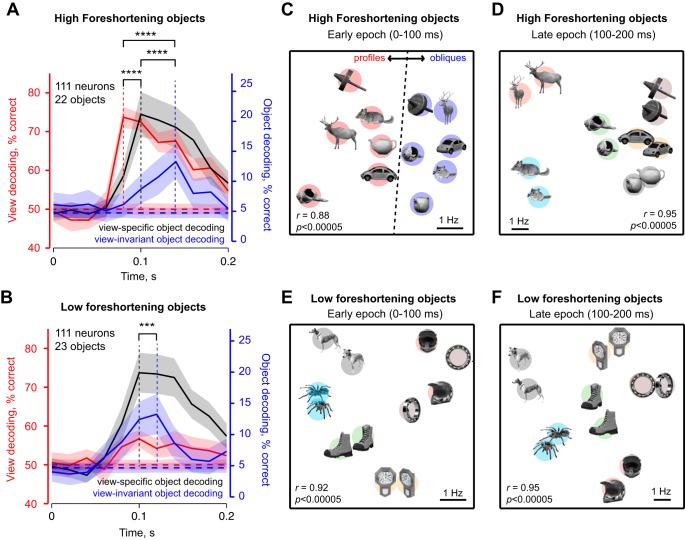
Dynamics of population decoding of view and object identity. *A*: trial-by-trial decoding accuracy for view (red), view-specific object identity (gray) and view-invariant object identity (blue) for high-foreshortening objects (*n* = 22). The lightly shaded regions depict the performance (mean and standard deviation) for a shuffle control in which the same analyses was repeated with object labels permuted randomly across trials. *B*: analogous plot for low-foreshortening objects. *C*: visualization of similarity relations in the early part of the visual response for a subset of high-foreshortening objects as obtained using multidimensional scaling. In the plot, images that elicited similar activity across the population are closer. *D*: similarity relations in the late part of the visual response for high-foreshortening objects. *E* and *F*: analogous plots for low-foreshortening objects. [Object pictures from Amsterdam Library of Object Images (Geusebroek et al. 2005), reprinted with permission; or Hemera Photo Objects, reprinted with permission, copyright 2015, S. P. Arun and licensors, all rights reserved.]

We compared decoding dynamics for two groups of objects: objects with high (*n* = 22) and low foreshortening (*n* = 23). For high-foreshortening objects, view decoding accuracy peaked early in the visual response (*t* = 80 ms after stimulus onset), followed 20 ms later by view-specific object decoding, and then 40 ms later by view-invariant object decoding (Fig. 5*A*). View-specific object decoding was always stronger than view-invariant object decoding, indicating that there was only partial generalization across viewpoint in IT neurons. To assess the statistical significance of these latency differences, we performed a bootstrap analysis. We repeatedly selected 111 neurons chosen randomly with replacement and calculated the peak latency of view decoding, view-specific and view-invariant object decoding. Among the bootstrap latency estimates, 97% of them exhibited a longer latency for peak view-invariant decoding compared with peak view decoding, 83% showed a longer latency for peak view-invariant decoding compared with view-specific decoding, and 83% showed a longer latency for peak view-specific decoding compared with view decoding. The number of bootstrap samples was chosen here and throughout to be the number of data points (i.e., number of neurons), but we obtained qualitatively similar results on varying these specific choices. All latency comparisons were statistically significant: peak latency of view decoding was significantly smaller than the peak latency of view-specific object decoding, which was in turn smaller than the peak latency of view-invariant object decoding (*P* < 0.0005, Wilcoxon signed-rank test on 111 bootstrap latency estimates for all comparisons). Thus, for high-foreshortening objects, IT neurons initially encode view and view-specific object information and only later encode object identity invariant of view.

The fact that view decoding peaked earlier compared with view-specific object decoding might have been simply because the former had to distinguish between two alternatives (profile vs. oblique) and the latter between 22 alternatives (i.e., the 22 objects). To confirm that this was not the case, we repeated the same analysis on pairs of objects so that chance performance was 50% for all three classifiers: view decoder and the view-specific and view-invariant object decoders. Here too we obtained similar results: view decoding peaked earliest, followed by view-specific decoding, and then by view-invariant object decoding.

Decoding dynamics were qualitatively different for low-foreshortening objects (Fig. 5*B*). Here, view information was relatively weak and attained a peak at the same time as view-specific object information (*t* = 100 ms), but view-invariant encoding of object identity attained a peak 20 ms later (Fig. 5*B*). We performed a bootstrap analysis to determine the statistical significance of the latency differences. Across the samples chosen, 79% of them had a longer latency for view-invariant decoding compared with view specific and view decoding. The difference in latency between view-invariant object decoding and the other two decoders was statistically significant (*P* < 0.0005, Wilcoxon signed-rank test on 111 bootstrap-derived peak latency estimates).

If the mechanisms underlying view invariance require compensating for the response modulation due to changes in view, then strong view dependence should delay the onset of view invariance, and the magnitude of view dependence and view invariance should be negatively correlated. A closer inspection of the decoding dynamics for high- and low-foreshortening objects shows that this might indeed be the case: view-invariant decoding peaked slightly later for high-foreshortening objects where view decoding is stronger, compared with low-foreshortening objects (Fig. 5, *A* vs. *B*). To further test this prediction, we calculated correlation between peak view decoding and peak view-invariant object decoding across random groups of objects. We chose 5,000 random groups of 10 objects, each as a representative random sample of the full set of ^45^C_10_ (∼3 × 10^9^) possible groups. This yielded a significant negative correlation (*r* = −0.30, *P* < 0.00005). This correlation was robust: it was statistically significant even for as few as 200 random groups of objects, remained so even on varying the number of objects in each group and was abolished upon randomly shuffling view and object labels. We observed no such systematic dependence between view decoding and view-specific object decoding (*r* = 0.1, *P* = 0.14). We conclude that strong view modulation delays the onset of view-invariant signals in IT.

To visualize this transition in the neuronal representation, we performed a multidimensional scaling analysis on the IT neural population. For each pair of images, we calculated their dissimilarity according to the IT neural activity as the summed absolute difference in the firing rates. In the resulting plots, nearby images elicited similar activity across neurons. We performed this analysis for the early (0–100 ms) and late (100–200 ms) epochs in the visual response to capture the early sensitivity to view and the later encoding of object identity observed in Fig. 5*A*. For high-foreshortening objects, we observed a dramatic transition whereby profile and oblique views form distinct clusters (Fig. 5*C*) in the early part of the response, whereas views of the objects clustered together in the later part of the response (Fig. 5*D*). In contrast, for low-foreshortening objects, there was no such transition (Fig. 5, *E* and *F*).

### Experiment 2: Objects at Multiple Views

**Fig. 6.**
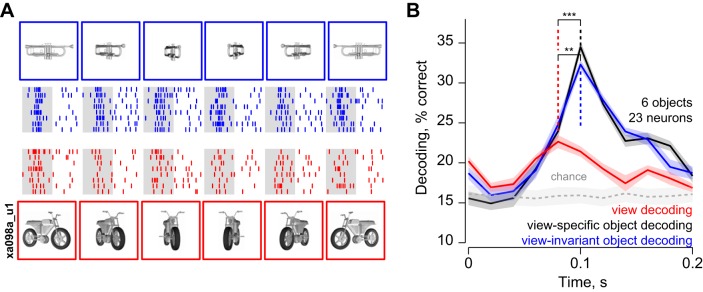
Dynamics of decoding for multiple views. *A*: example neuronal responses to two objects (trumpet and motorbike) for multiple views (*experiment 3*). Blue rasters represent spikes elicited by views of the trumpet; red rasters represent spikes evoked by views of the motorbike. *B*: dynamics of population decoding of view (red), view-specific (black) and view-invariant (blue) object identity across 6 objects and 6 views. In all panels, shaded regions represent bootstrap-derived estimates of standard deviation. The light gray shaded region represents the performance (mean and standard deviation) of a shuffle control in which the analyses were repeated with object labels permuted randomly. [3D models created by D. Buck (trumpet) and M. Rehman (motorbike). Reprinted with permission.]

The above results are based on neuronal responses to objects at two views. Do these results hold for objects at multiple views? To address this question, we recorded the responses of a small set of neurons (*n* = 23) to six high-foreshortening objects presented in six views each. Figure 6*A* shows the responses of an example IT neuron to two objects (trumpet and motorbike) across multiple views. This neuron responded strongly to all views of the trumpet and weakly to all views of the motorbike. This pattern was true across the population: neuronal responses to the six objects were correlated across every pair of views tested (average pairwise correlation across views: *r* = 0.37 across 21 neurons and ^6^C_2_ pairs of views; average significant correlation: *r* = 0.84).

We then measured the ability of this small population of neurons to signal viewpoint, view-specific and view-invariant object identity using population decoding as before. Concordant with the results of *experiment 1*, we found that decoding of views reached a peak slightly earlier than that of view-specific and view-invariant object identity (peak times: 80 ms for view, 100 ms for view-specific and view-invariant object decoding; Fig. 6*B*). The peak latency of view decoding was significantly earlier than the peak latency of view-specific and view-invariant object decoding, using a bootstrap analysis as before (*P* < 0.0005, Wilcoxon signed-rank test on 23 bootstrap-derived estimates of peak latency). Additionally, 87% of the bootstrap samples showed a later peak for view-specific and view-invariant decoding compared with view decoding. However, unlike in *experiment 1*, view-specific and view-invariant decoding attained a peak simultaneously; this is most likely because this latency difference is smaller and therefore harder to detect with the relatively small sample of neurons recorded in this experiment. We conclude that, for high-foreshortening objects, IT neurons undergo a transition from early viewpoint sensitivity to later encoding of view-invariant object identity, even for multiple views.

### Experiment 3: Silhouettes

Does the view invariance in IT depend on three-dimensional (3D) shape? To address this question, we designed an additional experiment based on the observation that reducing objects to their silhouette form reduces their 3D percept (Wagemans et al. 2008). We reasoned that, if view invariance depends on extracting 3D structure, it should be reduced in magnitude for object silhouettes. Furthermore, we observed that elongated planar objects are more likely to have distinctive features along their external contour, whereas rounder nonplanar objects are more likely to have distinctive internal features. Thus the silhouette of an elongated object is likely to contain the same distinctive features across views, whereas the silhouette of a round object can potentially contain very different features across views. We therefore predicted that view invariance for elongated (high-foreshortening) objects should be less affected by silhouetting compared with rounder (low-foreshortening) objects.

**Fig. 7.**
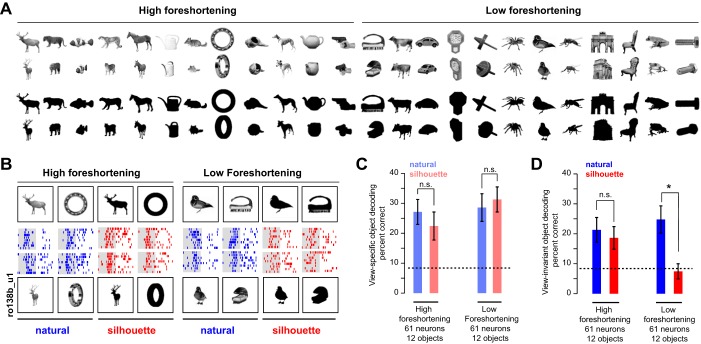
View invariance for silhouette objects (*experiment 3*). *A*: the full stimulus set used in this experiment, showing natural (*top* two rows) and silhouetted versions (*bottom* two rows) of both profile and oblique views. Silhouettes in the actual experiment were white against a black background. *B*: example responses of an IT neuron to profile and oblique views for high- and low-foreshortening objects, for natural and silhouette versions. *C*: view-specific object decoding accuracy (during 100–200 ms) for natural (blue) and silhouette (red) versions for high- and low-foreshortening objects. Error bars represent bootstrap-derived estimates of standard deviation. *D*: analogous plots for view-invariant object decoding. *Statistical significance (*P* < 0.05, rank-sum test on correct class labels across trials). [Object pictures from Amsterdam Library of Object Images (Geusebroek et al. 2005), reprinted with permission; or Hemera Photo Objects, reprinted with permission, copyright 2015, S. P. Arun and licensors, all rights reserved.]

To assess these possibilities, we recorded the responses of 61 IT neurons to a subset of 24 objects from *experiment 1* at both views in their natural and silhouette versions (Fig. 7*A*). The responses of an example neuron to natural and silhouette versions of a few objects are shown in Fig. 7*B*. It can be seen that the neuron responds similarly to natural and silhouette versions of high-foreshortening objects, but this pattern did not hold for low-foreshortening objects. This pattern was true across the neuronal population: view invariance, as measured by the profile-oblique response correlation, was comparable in magnitude for natural and silhouette versions for high-foreshortening objects (average significant correlations: *r* = 0.51 and 0.52 for naturals and silhouettes, *P* = 0.96, Wilcoxon rank-sum test; average correlations: *r* = 0.20 and 0.24). In contrast, for low-foreshortening objects, there was a drop in view invariance that did not attain statistical significance (average significant correlations: *r* = 0.70 and 0.54 for naturals and silhouettes, *P* = 0.74, Wilcoxon rank-sum test; average correlations: *r* = 0.22 and 0.24).

To further confirm this trend, we repeated the object decoding analysis as before: we trained separate linear classifiers on responses to natural and silhouette versions of object and compared view-specific and view-invariant object decoding accuracy in the later epoch of the visual response (100–200 ms). We observed no significant difference in view-specific object decoding between natural and silhouette versions for both groups of objects (Fig. 7*C*). Thus, despite silhouetting, images were equally distinct from each other at each view and could be decoded just as easily. However, there were systematic changes in view-invariant object decoding due to silhouetting. For high-foreshortening objects, there was no reduction in decoding accuracy, suggesting that even the outer contours of objects are sufficient for view invariance (Fig. 7*D*). In contrast, for low-foreshortening objects, we observed a dramatic drop in object decoding (Fig. 7*D*). We conclude that silhouetting does not diminish view invariance in IT neurons as long as it preserves distinctive contour features across views.

### Computational Models of View Dependence and Invariance

In the preceding sections, we have shown that, for objects that foreshorten across views, IT neurons exhibit a dynamic transition from early sensitivity to viewpoint, to later view-invariant encoding of object identity. To investigate the possible underlying computations, we tested a number of computational models for their ability to capture the early view dependence and late view invariance in the IT population.

To compare model and IT representations on the same footing, we devised a measure of clustering strength which captures the degree to which views or objects cluster together. This clustering strength measure is simply the ratio d_between_/(d_between_ + d_within_), where d_between_ is the average distance between clusters and d_within_ is the average distance within a cluster. In general, this clustering strength measure can range from 0.5 (if distances are random) to 1 (for strong clustering). We calculated two clustering strength measures: one for the tendency of views to cluster together (to capture the early view dependence in IT) and one for the tendency of objects to cluster together (to capture the late view invariance in IT). For IT neurons, we calculated the distance between each pair of images as the absolute difference in firing rates evoked by the images, averaged across all neurons. For models, we calculated the average absolute difference between the feature vectors corresponding to the images. To assess models for their ability to capture the early view invariance in IT (i.e., during 0–100 ms after image onset), we asked whether the degree of view clustering covaried with the view clustering observed in IT across many random groups of objects. Likewise, to assess models for their ability to capture the late object clustering in IT (i.e., during 100–200 ms after image onset), we asked whether the degree of object clustering covaried with the object clustering observed in IT across random groups of objects.

**Fig. 8.**
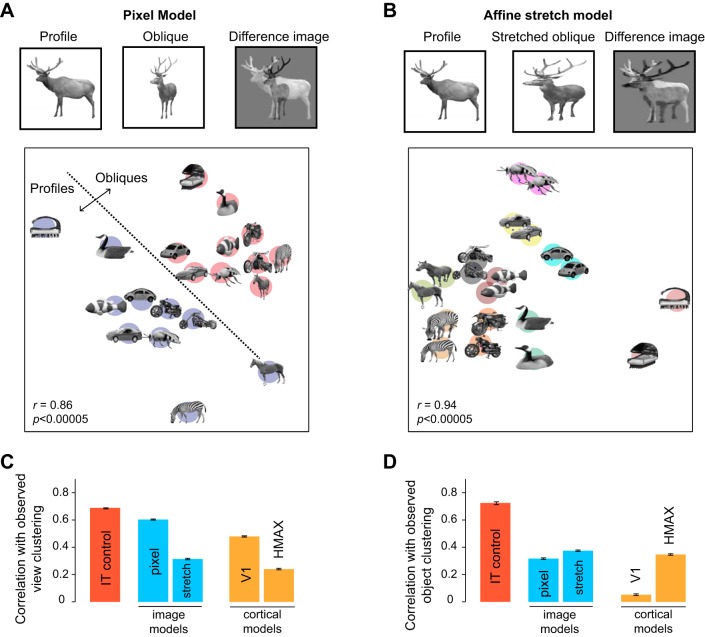
Computational models of view dependence and invariance. *A*, *top*: schematic of the pixel model. The distances between a pair of images in the pixel model was calculated by normalizing each image so that the sum of pixel intensities is 1, and then calculating the sum of the absolute difference between the pixels of the two images (*right*). For the pixel model, there is a large distance between the profile and oblique views, especially for high-foreshortening objects. *Bottom*: a multidimensional scaling plot to visualize distances between a few of the objects. Distances between each pair images is proportional to their pixel distance (*r* = 0.86 between the original distances and distances in this two-dimensional plot). It can be seen that the object representation in the pixel model is highly dependent on view. This plot is identical to Fig. 4*B*. *B*, *top*: schematic of the affine stretch model. The profile and oblique views of each object were equated in size by stretching. This stretching was equivalent for most objects to an affine matching transformation between the two views. After this step, the distance between each pair of images was calculated exactly as in the pixel model. *Bottom*: a multidimensional scaling plot of the object representation in the stretch model. It can be seen that the stretch model gives rise to a view-invariant representation. *C*: correlation between observed early view clustering in IT with view clustering in the pixel, stretch, V1 and HMAX models, calculated across 10,000 random groups of 10 objects each. The IT control was calculated as the correlation in view clustering between two random halves of the neuronal population with a Spearman-Brown split-half correction. This IT control represents the degree to which the IT data are self-consistent and are an upper bound on model performance. *D*: correlation between observed late object clustering in IT and model object clustering for all four models. The IT control is calculated as before, except using object clustering. [Object pictures from Amsterdam Library of Object Images (Geusebroek et al. 2005), reprinted with permission; or Hemera Photo Objects, reprinted with permission, copyright 2015, S. P. Arun and licensors, all rights reserved.]

We tested four models for their ability to account for the IT invariance dynamics. The first two models were based on simple computations performed directly on the image. The first model is the pixel model described earlier: in this model, distances between images are simply the sum of absolute differences in pixels (Fig. 8*A*). For high-foreshortening objects, this model produces view dependence; this can be seen in a multidimensional scaling plot performed on a subset of the objects (Fig. 8*A*). The second model instantiated an affine matching computation that stretched oblique views to match the width of the corresponding profile view (Fig. 8*B*). This affine stretching operation is optimal for planar objects. This model produces view-invariant clusters as illustrated in a multidimensional scaling analysis performed on the same set of objects as before (Fig. 8*B*). The next two models were widely used computational models of visual cortex (see methods): a model of V1 neurons (Pinto et al. 2008) and the HMAX model (Riesenhuber and Poggio 2000).

The performance of all four models is summarized in Fig. 8, *C* and *D*. Across random groups of objects, the view dependence in IT was predicted best by the pixel and V1 models (*r* = 0.6 and 0.48 for the pixel and V1 models, *P* < 0.0005, calculated across 100,000 random groups of 10 objects each; Fig. 8*C*). In contrast, the affine stretch model and the HMAX model did not capture the variation in view dependence (*r* = 0.31 and 0.24 for the stretch and HMAX models, *P* < 0.0005). Thus low-level pixel differences or V1-like units explain the early view dependence in IT.

Across random groups of objects, the later view invariance in IT was predicted poorly by the V1 model (*r* = 0.05, *P* > 0.05 for the V1 model; Fig. 8*D*). It was predicted better by the pixel model (*r* = 0.31, *P* < 0.0005), but best by the affine stretch and HMAX models (*r* = 0.38 and 0.35 for the affine and HMAX models, *P* < 0.0005; Fig. 8*D*). Since the affine and HMAX models yield equivalent fits, our data cannot reliably distinguish between them. However, of the two models, the affine model provides a more parsimonious account of view invariance in IT, given our data.

Could the low correlation between the model-predicted and observed view invariance be because the models did not account for object-object dissimilarities at each view? To assess this possibility, we compared dissimilarities between objects in their profile view obtained from each model with those observed in IT. All models yielded comparable and low correlations with IT distances (correlation between ^45^C_2_ model and IT distances for objects in their profile view: *r* = 0.30, 0.32, 0.41 and 0.27 for the pixel, stretch, V1 and HMAX models respectively, *P* < 0.00005).

### Do Dynamics of View Invariance Differ from That of Size Invariance?

**Fig. 9.**
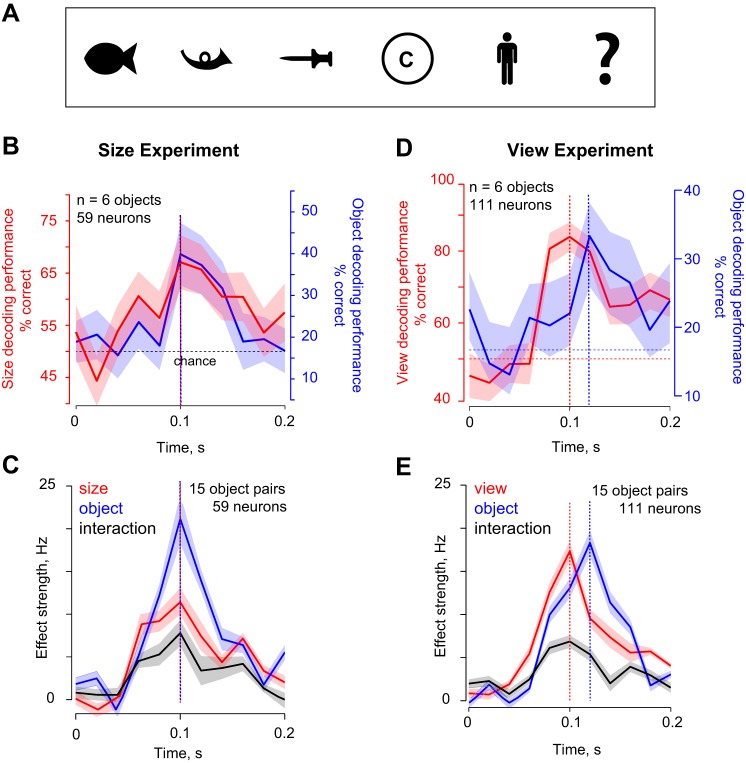
Dynamics of size invariance in IT. *A*: stimuli used in the size experiment. Each stimulus was presented at two sizes (3° and 6°). *B*: size and object decoding dynamics in 20-ms bins showing that size-invariant object decoding (blue) and size decoding (red) accuracies both peak at 100 ms. The red and blue vertical lines indicate the times at which decoding accuracies reached a peak, and the dotted horizontal line indicates chance performance. *C*: size, object and interaction effect strengths in 20-ms steps, showing no dynamic transition for size invariance. *D*: view (red) and view-invariant object decoding (blue) dynamics for the 6 highest foreshortening objects in the view experiment (*experiment 1*), whose baseline invariance index was negative and roughly in the same range as in the size experiment (mean baseline index for all 15 object pairs: −0.03 for size experiment, −0.1 for the view experiment). *E*: view, object and interaction effect strength dynamics for the same objects.

Are the dynamics of view invariance different compared with other identity-preserving transformations? Although addressing this question systematically is beyond the scope of this study, we obtained some insight using existing data in our laboratory (Vighneshvel and Arun 2015) in which we had recorded the responses of 59 IT neurons to 6 shapes (Fig. 9*A*), presented at two retinal sizes (3° and 6°). Using these data, we calculated the decoding dynamics as before for size decoding and size-invariant object decoding. From the view experiments, we predicted a similar dynamic transition for objects with large changes in size compared with changes in object identity. However, we observed no such transition: instead, size and object identity decoding had similar dynamics across the visual response (Fig. 9, *B* and *C*). The dynamics were entirely different for view invariance, even when we selected objects with roughly equal modulation levels to the objects in the size experiment (Fig. 9, *D* and *E*). The fact that size and object identity have similar dynamics is consistent with previous reports that larger stimuli evoke consistently larger responses throughout the visual response (Ito et al. 1995; Sripati and Olson 2009). These data suggest that the dynamics of view invariance are potentially different from other identity-preserving transformations. However, because the size and view experiments involved different neural populations and image sets, we cannot rule out these differences as an explanation for the different dynamics. Testing this possibility will require measuring the dynamics of several types of invariance while equating image changes across identity-preserving transformations.

## DISCUSSION

Here, we characterized the dynamics of 3D view invariance in monkey IT neurons for naturalistic objects. The main finding of our study is that IT neurons exhibit a dynamic transition in their representation, whereby they are initially viewpoint sensitive and only later become view invariant. This transition occurred for objects that foreshortened strongly across views. In general, the dynamics depended on two specific image attributes: foreshortening and dissimilarity. View invariance was present even in silhouettes, suggesting that it can arise even through there is similarity between external contours across views.

The high view invariance observed here for naturalistic objects in naive monkeys stands in contrast to the extreme view dependence observed in IT neurons for artificial objects, even after extensive training (Logothetis et al. 1994, 1995; Logothetis and Pauls 1995; Yamane et al. 2008). This is most likely due to differences in object structure (Biederman 2000; Biederman and Gerhardstein 1993): images of a natural object vary systematically across rotations in depth, whereas images of wireframes change drastically. It might also arise because artificial objects can be perceptually highly similar to each other, resulting in view dependence (Edelman 1995; Foster and Gilson 2002; Newell 1998; Wilson and Farah 2003). Our finding that IT neurons are view invariant for naturalistic objects is consistent with the view invariance observed previously for familiar natural objects and faces (Booth and Rolls 1998; Perrett et al. 1991). Our results suggest that the visual system is optimized not just for the features present in natural objects, but also for how these features change with viewpoint.

The most striking finding of our study is the transition in IT neurons from an early encoding of view and view-specific object information to a later encoding of view-invariant object identity. This transition cannot be explained by uncontrolled but systematic differences present in our choice of objects or views. Suppose, for instance, changes in object identity produced larger image changes compared with views (as they indeed did, see Fig. 4). This predicts stronger object modulation throughout the response, but not a transition. Conversely, if views caused larger image changes than object identity (as they do for high-foreshortening objects), this predicts stronger view modulation throughout, but not a transition. Similarly, this transition cannot be explained by the finding that IT neurons are more sensitive to nonaccidental properties than metric properties (Kayaert et al. 2003), because this would predict a fixed effect throughout time, but not a transition. Thus this transition cannot be explained by any fixed differential sensitivity of neurons to views or objects.

To our knowledge, no previous study has examined the dynamics of view invariance over the time course of neuronal responses in IT. The transition from view dependence to invariance reported here has interesting parallels in the macaque face-processing literature. Previous studies have reported view-dependent representations for faces in the anterior inferior temporal gyrus, but invariant representations in the anterior STS (Eifuku et al. 2004, 2011). More recently, it has been reported that neurons in posterior face patches show view-dependent responses to faces, whereas anterior face-patch neurons encode view-invariant face identity (Freiwald and Tsao 2010). However, these studies have not investigated the dynamics of view-invariant representations as we have done here. In the framework used in our study, faces foreshorten relatively little across views, but tend to be similar in their pixels. This greater similarity between individual faces predicts a later onset of view-invariant signals for faces. We speculate that neurons in anterior STS or anterior face patches exhibit a transition similar to that reported here, wherein they are initially modulated by face view and only later show view-invariant encoding. Viewpoint generalization might thus involve similar computations for faces and objects.

The dynamics of view invariance observed here are consistent with reports that IT neurons signal coarse, global information sooner than finer, local information (Sugase et al. 1999; Sripati and Olson 2009). In this context, the early availability of object view information may reflect spatial scale or aspect ratio rather than object viewpoint per se; this corroborates our finding that low-level properties such as foreshortening (Fig. 3*E*) and simple pixel models (Fig. 8) explain the observed view dependence. Even in the human vision literature, view dependence is considered a low-level property (Demeyer et al. 2007; Tjan and Legge 1998). In general, the early visual response in IT may be sensitive to low-level properties, such as size, position, viewpoint and contrast (Bar 2001; Rust and Dicarlo 2010, 2012) and may be inherited from early visual areas. The later portion of the response may reflect invariant signals and may involve nonlinear interactions between features (Brincat and Connor 2006; McMahon and Olson 2009; Sripati and Olson 2010). Thus the dynamics of view invariance observed here might reflect a general pattern of increasing invariance to low-level image properties over the course of the visual response in IT.

There are several theories for how view invariance might arise in the brain (Ghose and Liu 2013; Perrett et al. 1998; Shepard and Metzler 1971). First, it could arise trivially if an object has features diagnostic for its recognition, such as a distinctive color or texture (Biederman and Gerhardstein 1993; Hayward and Williams 2000; Tarr and Buelthoff 1995). Our finding that view-invariant object signals in IT arise later can be interpreted as encoding of distinctive texture information across views. However, we consider this unlikely because reducing objects to silhouettes (thereby removing texture) did not abolish view invariance (Fig. 7*D*). This finding also indicates that view-invariant responses can be driven by similarity between external contours across views. Indeed, IT neurons are sensitive to both features along external contours (Brincat and Connor 2004, 2006) as well as to internal 3D features (Yamane et al. 2008). Both types of information might be integrated to produce viewpoint invariance.

A second possibility is that view invariance could arise through extraction of view-invariant features: for instance, the exact angle between two lines varies with view, but the presence of a corner does not (Biederman 2000). Third, it could arise through associations between views learned during natural vision (Li and DiCarlo 2008; Riesenhuber and Poggio 2000; Wallis and Buelthoff 1999), although this by itself does not explain how neurons are view invariant for novel objects. There is some evidence that view invariance increases as novel objects become familiar and are seen while rotating continuously (Wallis and Buelthoff 2001). This may be because observers learn to form view-invariant representations of the entire object, or alternatively because they learn to identify familiar parts or features that have invariant representation. Our results suggest that view invariance can partially be explained using an affine matching between views (Fig. 8), and that it is present even for silhouettes (Fig. 7). These observations, together with the dynamics of view invariance and their dependence on object structure, place important constraints on theories of object recognition. Ultimately, a full account of view invariance will involve understanding feature representations along the ventral pathway (Connor et al. 2007), as well as their interplay with invariance (Dicarlo et al. 2012).

Our finding that view invariance in IT neurons depends on object structure is concordant with similar observations in human vision (Andresen et al. 2009; Biederman and Cooper 1991; Biederman and Gerhardstein 1993, 1995; Fang and He 2005; Gauthier et al. 2002; Hayward and Tarr 1997; Tarr 1995; Tarr et al. 1997, 1998; Tarr and Buelthoff 1995). Our results elucidate the viewpoint debate in several ways: first and foremost, our finding that view dependence and view invariance are dynamically present within the same neuronal population helps reconcile the disparate findings in the literature (Foster and Gilson 2002; Stankiewicz 2002). It is consistent with the finding that invariant representations develop dynamically in human magnetoencephalography data (Isik et al. 2013). Second, we have identified specific image properties (foreshortening and dissimilarity) that govern these dynamics. Third, we have shown that view invariance is present even when objects are reduced to silhouettes, suggesting that it does not require the extraction of 3D structure, but instead can arise because of similarity in the external contours across views. Taken together, our results suggest that view dependence or invariance can both manifest in behavior, depending on object structure and on the underlying epoch in the neuronal response.

## GRANTS

This study was supported by an Intermediate Fellowship from the Wellcome Trust-Department of Biotechnology (DBT) India Alliance, DBT-Indian Institute of Science partnership programme, a startup grant from the Indian Institute of Science (to S. P. Arun), and a Senior Research Fellowship from the Council for Scientific and Industrial Research, Government of India (to N. A. Ratan Murty).

## DISCLOSURES

No conflicts of interest, financial or otherwise, are declared by the author(s).

## AUTHOR CONTRIBUTIONS

Author contributions: N.A.R.M. and S.P.A. conception and design of research; N.A.R.M. and S.P.A. performed experiments; N.A.R.M. and S.P.A. analyzed data; N.A.R.M. and S.P.A. interpreted results of experiments; N.A.R.M. and S.P.A. prepared figures; N.A.R.M. and S.P.A. drafted manuscript; N.A.R.M. and S.P.A. edited and revised manuscript; N.A.R.M. and S.P.A. approved final version of manuscript.
